# Conservatism in Railway Surgery

**Published:** 1908-10

**Authors:** W. P. Whittington

**Affiliations:** Asheville, N. C.


### CONSERVATISM IN RAILWAY SURGERY.

BY W. P. WHITTINGTON, M. D., ASHEVILLE, N. C.

In presenting this paper, I do not wish to do so in a spirit of criticism, but wish to say something that will be of benefit to the Surgeon both civil and railway, and that will possibly be the means of saving the life or a useful limb of some unfortunate victim of a railway accident.

The position of a railway surgeon is a dual one; professional and diplomatical. His professional duty would require him to consider specifically the relief and safety of his patient, while as a railway official he must take into consideration the interests of the company by which he is employed. In reality he should and must have at heart the mutual interest of both. While this is very desirable it is still a hard matter to eliminate the influence of the employer over the employed and do justice both to the railway and the injured. The powers and influence of the surgeon over public opinion, is such that he may easily favor his employer and at thee sametime make it appeear that he has done the best possible for the injured.

For instance, the surgeon may be called to a patient with a mangled hand or foot, that looks to the layman to be beyond repair, or out of the power of the surgeon to save. The surgeon knows that if the limb could be saved it would require a long, tedious watching, and careful antiseptic treatment. He also knows that if he amputates above the injury he has healthy tissue, a clean stump and that in two or three weeks the patient is well, but minus a member that might have been saved and have been of great benefit to the injured.

In such a case the surgeon is commended for doing the best thing posible for his patient. The patient is furnished an artificial leg, or given a small compensation for a hand and goes through life maimed and worth but little to himself and family; when if he had been treated antiseptically this limb would have been saved and would have been a great blessing to himself and family.

I know a man who had a badly lacerated hand and his surgeon told him it must be amputated. The patient refused to have it done. The surgeon told him that he would have to put him to sleep before he could dress it. The patient submitted to the anaesthesia, but told the surgeon that if he amputated the hand he would kill him. The hand got well and the man can do any kind of work. He has a crippled but useful hand.

I know a man who had a compound comminuted fracture of the leg, opening up the ankle joint. It looked like an impossibility to save the foot. The foot was wrenched to one side so that the ankle was dislocated and the end of the tibia protruded through the skin. A drainage tube was passed clear through the joint, and the leg was treated antiseptically. The man got well with a useful foot and leg, but with a limited amount of motion. I could report a great many cases of extensive laceration, fractures, contusions and mangled conditions that have been saved by patient and persevering antiseptic and supportive treatment. While on the other hand I have seen valuable limbs sacrificer for want of such treatment. One might say that it is best not to subject our patients to so great a risk as is necessary to carry out an expectant antiseptic course of treatment. That there is danger of sepsis, gangrene and death. That there is always some danger we will admit. The surgeon should be very alert and when bad symptoms appear he should pursue such methods as is necessary to remedy the threatened evil. We are not justified in amputating a foot because a nail has pricked the heel and we are afraid the patient might have tetanus.

The method to pursue in these cases of railway and other injuries to the extremities is one of thorough antisepsis from the beginning. The patient should be cleansed as thoroughly as possible without further contaminating the wound. The injured limb should then be thoroughly washed with soap and water, bichloride of mercury one in two to four thousand, owing to the extent of the injury. This should be followed with alcohol and ether and finally rinsed with bichloride. In extensive injuries where there is a great deal of denuded tissue, it is best to continue a wet dressing of a satured solution of boracic acid or sulphate of alum. In very extensively mangled legs or arms the limb may be immersed for from twenty-four to seventy-two hours in a saturated solution of sulphate of alum. This solution of sulphate of alum is not extensively used, so far as I am informed, but it is practically harmless and has fine antiseptic powers and acts as an astringent on the extensively lacerated and bleeding tissues. As soon as the bleeding ceases and if the tissues show an anaemic appearance the solution should be discontinued or made weaker. Dry or moist antiseptic dressing should be continued and sloughing surfaces separated as early as possible, care being taken not to open up raw surfaces more than is necessary, as such a proceeding would encourage the absorption of septic matter. Another good method is after the parts are thoroughly cleansed with soap and water, to mop the raw surface thoroughly with pure carbolic acid followed with 95 per cent alcohol, and paint it thoroughly with tincture of iodine. After healing has taken place it may be necessary to do plastic operations to complete the work that nature failed to do.

